# Prevalence and predictors of workplace violence against nurses in Africa: A systematic review and meta‐analysis

**DOI:** 10.1002/hsr2.2068

**Published:** 2024-04-21

**Authors:** Emmanuel Ekpor, Emmanuel Kobiah, Samuel Akyirem

**Affiliations:** ^1^ School of Nursing and Midwifery University of Ghana Accra Ghana; ^2^ Christian Health Association of Ghana Accra Ghana; ^3^ Yale School of Nursing Yale University New Haven Connecticut USA

**Keywords:** abuse, Africa, harassments, nurse, threat, workplace violence

## Abstract

**Background and Aims:**

Workplace violence (WPV) against nurses is a pervasive global issue, yet the extent of this phenomenon in the African context remains insufficiently explored. This review aimed to synthesize the available literature to identify the prevalence and predictors of WPV against nurses in Africa.

**Methods:**

A systematic search was conducted across MEDLINE, CINAHL, PsycINFO, and Scopus, to identify studies published from 2000 to October 2023. The pooled prevalence of WPV and it subtypes were estimated using random‐effect meta‐analysis. Heterogeneity between studies was quantified with *I* ^2^ statistics. Subgroup analysis and meta‐regression were performed to identify sources of heterogeneity.

**Results:**

This review included 27 studies, involving 9831 nurses. The pooled prevalence of WPV was 62.3% (95% confidence interval [CI]: 51.6–72.0). Verbal abuse emerged as the most common form of WPV, with a prevalence rate of 51.2% (95% CI: 41.3–61.1), followed by threat 23.3% (95% CI: 6.5–57.2), bullying 22.9% (95% CI: 14.0–35.2), physical abuse 15.1% (95% CI: 11.0–20.4), and sexual harassment 10.3% (95% CI: 5.9–17. 5). The proportion of WPV varied across geographical areas in Africa; however, the differences were not significant. The predictors of WPV encompassed demographic factors, personal habits, workplace characteristics, and nurses’ past experience.

**Conclusion:**

WPV against nurses is prevalent in Africa and transcends geographical boundaries in this region. This underscores the urgent need for targeted interventions and policy changes to address this issue in Africa.

## 1 INTRODUCTION

Workplace violence (WPV) is a pervasive and alarming issue that transcends geographical boundaries. This phenomenon is defined as “incidents where staff are abused, threatened, or assaulted in circumstances related to their work.”^1^ WPV is particularly prevalent in the healthcare settings, constituting a quarter of all violent cases in the workplace.^2^ The World Health Organization reports that up to 38% of healthcare workers experience physical violence at some point during their careers.^3^ Even more disconcerting, a recent systematic review indicates that as high as 61.9% of healthcare workers have encountered some form of WPV, including physical assault, aggression, sexual harassment, bullying, verbal abuse or threats.^4^ These figures, alarming as they are, might not even capture the full extent of the issue due to significant under‐reporting of violent incidents in the workplace.

Nurses, due to their direct patient interaction, are particularly vulnerable to WPV compared to other clinical staff.^5^ Shockingly, studies have highlighted that nurses so frequently endure violence that these incidents are often normalized as “part of the job.”^6^ A recent systematic review has indicated that up to 87% of female nurses reported experiencing sexual harassment.^7^ In Iran, a study found that 91% of nurses faced intimidation and bullying at their workplace,^8^ while in China, 79% of nurses were exposed to different forms of WPV.^9^ Studies have shown that the prevalence of WPV varies across various factors, such as gender, age, income, academic qualification, working experience, professional rank, and many others.^8^, ^9^

The issue of WPV against nurses has garnered increasing attention in recent years due to its profound impact on the healthcare sector. WPV, in its manifold manifestations, not only jeopardizes the safety of nurses but also affects the quality of care provided and organizational outcomes. Acts of violence in the workplace contribute to an atmosphere of fear and psychological stresss, consequently impacting the mental health of nurses.^6^, ^10^ A recent study has discerned that nurses who experience WPV are more likely to suffer from burnout, which in turn was associated with less job satisfaction.^11^ Another study has revealed that nurses who have experienced violence are at significantly higher risk of impaired work functioning.^12^ Additionally, WPV is associated with heightened turnover intentions among nurses, underscoring its significant ramifications on the sustainability of the healthcare workforce.^13^

Indeed, WPV has a detrimental impact on the quality of care provided, and this is particularly critical in the African continent, given the unique healthcare challenges in this region. Many African healthcare facilities operate with limited resources, including staffing levels. When healthcare workers are subjected to violence, it can exacerbate an already challenging working environment, leading to burnout and a decline in the quality of care. Despite the gravity of this issue, existing studies on the prevalence of WPV against nurses in Africa have not been synthesized, revealing a critical gap in our understanding of the extent of this phenomenon against nurses in this region. This review aimed to identify the prevalence and predictors of WPV against nurses in Africa. Addressing this gap is essential, as the findings of this study have implications for the implementation of preventive measures to protect nurses and improve the quality of healthcare services in Africa.

## 2 METHODS

The methodology for conducting systematic reviews of prevalence and cumulative incidence data, as outlined by the Joanna Briggs Institute (JBI), served as the guiding framework for this review.^14^ The findings of this review was reported in line with the preferred reporting items for systematic review and meta‐analysis (PRISMA). The protocol for this review was prospectively registered with PROSPERO (Registration number: CRD42023475587).

### 2.1 Inclusion and exclusion criteria

The following criteria were used for the included studies: (1) quantitative or mixed method studies that measured or reported the prevalence estimates of WPV and it types; (2) nurses as the target of WPV; (3) studies conducted in an African country (4) studies published from the year 2000 (5) published in the English language. We excluded review articles, qualitative studies, and articles in the grey literature (e.g., posters, conference abstracts). In cases where a single study was reported in multiple publications, only one study was selected based on its relevance to the outcome of interest.

### 2.2 Search strategy

A systematic systematic search was conducted on October 11, 2023, employing multiple databases including MEDLINE, CINAHL, PsycINFO, and Scopus. Additionally, we searched the Africa Journal Online (AJOL) and reviewed the reference lists of retrieved articles to identify any other pertinent studies. Using the condition, context, and population (CoCoPop) framework,^15^ our search strategy for the various databases encompassed the terms “workplace violence,” “Africa,” and “nurses.” Both controlled vocabulary and keywords to the search terms were incorporated, with the Boolean operators “OR” and “AND” applied appropriately. The search was limited to studies published from the year 2000 onwards. This was to ensure the inclusion of the most contemporary and pertinent research findings that accurately reflecting the present state of evidence on this topic. The full details of the search strategy are provided in Table S1.

### 2.3 Study selection

Duplicates from retrieved articles were removed using EndNote 20, after which the remaining articles were then uploaded to Rayyan^16^ for title and abstract screening. Subsequently, the full text of selected studies was retrieved and carefully assessed to ascertain their eligibility for inclusion in this review. Two authors (EE and EK) independently conducted the screening and selection of the articles meticulously. In the event of any discrepancies, a discussion with a third author (SA) was held to reach consensus.

### 2.4 Data extraction

We devised a standardized data extraction form in Excel format to retrieve relevant information from the included studies. The data gathered encompassed various aspects, including study‐specific attributes (such as the primary author's name, publication year, study location, research design, and sample size), participant demographics (average age, gender distribution), and our outcome of interest (prevalence and predictors of WPV). Additionally, any supplementary data that emerged and could enhance our analysis was meticulously recorded and integrated into our dataset.

### 2.5 Quality assessment

The JBI critical appraisal checklist for studies reporting prevalence data was utilized to assess the methodological quality of the included studies.^15^ This assessment tool comprises nine critical questions and offers response options of “Yes,” “No,” “Unclear,” and “Not applicable.” Without a predefined threshold for grading the quality of studies, we determined that studies that had 7–9 “yes”, 5–6 “yes” and less than 5 “yes” as having low, moderate, and high risk of bias, respectively. Full detail of the quality assessment tool is presented in the Table S2.

### 2.6 Data analysis

The prevalence estimate was pooled using a generalized linear mixed model with the logit transformation^17^ and the 95% confidence interval (CI) calculated using the Clopper‐Pearson interval. Given the potential for considerable variability among the studies included, we utilized the random‐effects model. We employed the *I* ^2^ statistic to quantify the proportion of variability due to heterogeneity across studies, with values of 25%, 50%, and 75% representing low, moderate, and high levels of heterogeneity respectively.^18^ Subgroup analysis was done by stratifying data by geographic area within Africa, sample size, number of facilities, year of study, duration of WPV estimate, and academic qualification of participant. Furthermore, we conducted meta‐regressions using a mixed‐effects model to investigate sources of heterogeneity among studies. The presence of publication bias was evaluated using funnel plots and was statistically explored using the Egger's test.^19^ The meta package in R statistical software was used for the meta‐analysis. Narrative synthesis was used to textually summarize the predictors of WPV, focusing on studies that reported the adjusted effect measure size of the associated factors.

## 3 RESULTS

### 3.1 Search results

**Figure 1:**
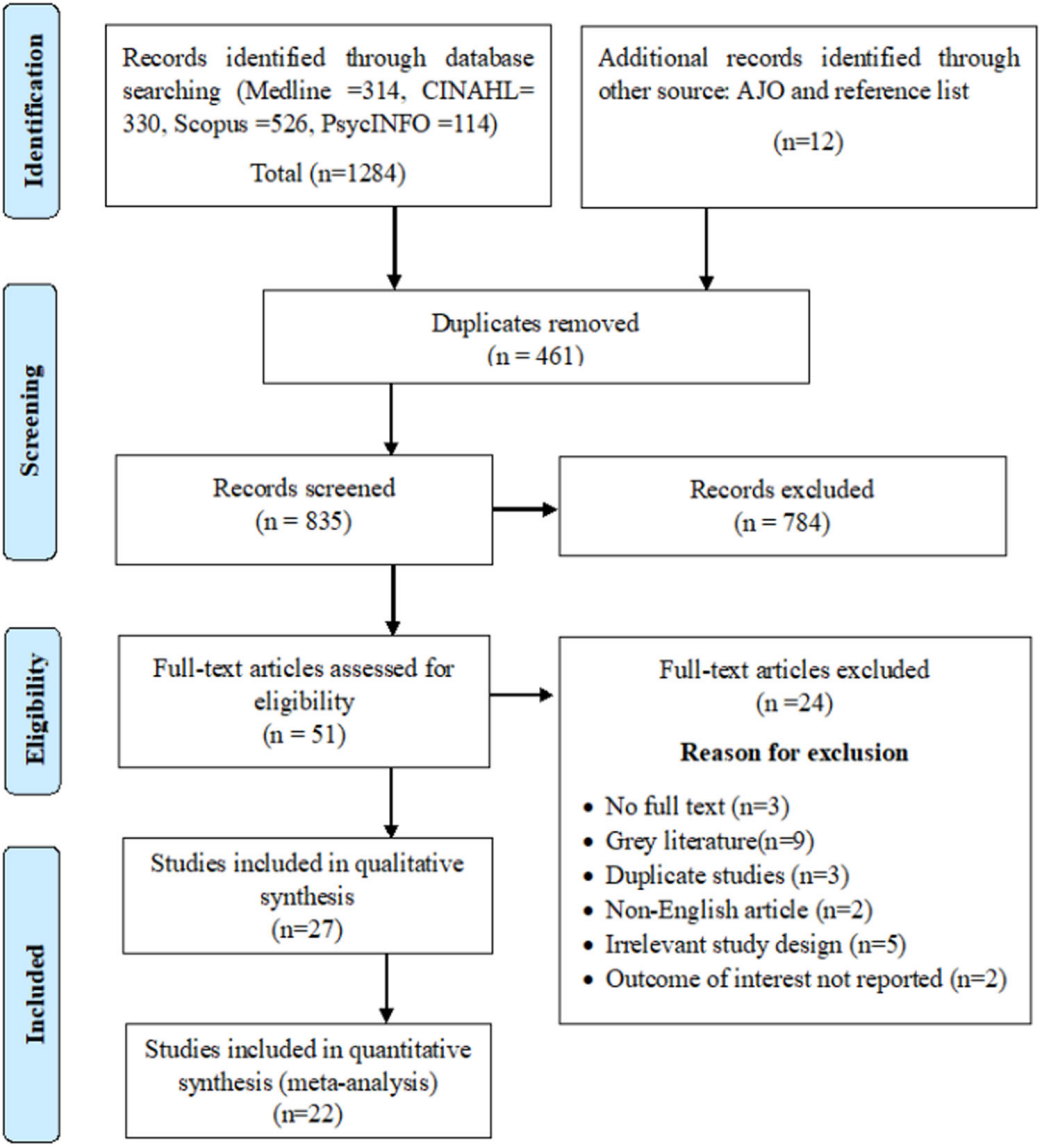
PRISMA flow chart summarizing the article selection process. PRISMA, reporting items for systematic review and meta‐analysis.

Our systematic search yielded a total of 1296 studies, consisting of 1284 records from four databases (MEDLINE, CINAHL, PsycINFO, and Scopus) in addition to 12 records from alternative sources. Following the removal of duplicate entries, we screened 835 articles based on their titles and abstracts, of which 51 articles were eligible for full‐text screening. Ultimately, 27 articles met our inclusion criteria for this study. A summary of the steps involved in the screening process and reasons for exclusion of articles after full‐text review are provided in Figure 1.

### 3.2 Characteristics of included studies

The studies incorporated in this review primarily employed cross‐sectional research designs, with only one study adopting a mixed‐method approach. These studies were published from the years 2005 to 2023, with a noteworthy proportion of them (*n* = 14) being published in the most recent 5 years. The total participation encompasses 9831 nurses, with sample sizes varying between 81 and 970. The majority of participants were females (66.9%) as per studies that reported both gender proportions. The mean age of the participants ranges from 27.7 to 40.9. The participants were recruited across 162 (numbers ranging from 1 to 23) health facilities. The studies were conducted in 10 different countries, with majority conducted in Ethiopia (*n* = 8),^20^, ^21^, ^22^, ^23^, ^24^, ^25^, ^26^, ^27^ followed by Egypt (*n* = 7),^28^, ^29^, ^30^, ^31^, ^32^, ^33^, ^34^ South Africa (*n* = 3),^35^, ^36^, ^37^ Ghana (*n* = 2),^38^, ^39^ Nigeria (*n* = 2),^40^, ^41^ and 1 each from Gambia,^42^ Malawi,^43^ Kenya,^44^ Rwanda,^45^ and Tanzania.^46^ The quality of the studies ranged from 3 to 9, with majority (*n* = 16) having low risk of bias (Table S2).

In relation to the measurement of WPV, the majority of the studies (*n* = 19) assessed the prevalence of WPV based on nurses’ experiences over the preceding 12 months. Three studies focused on a 6‐month time frame, while one study measured lifetime experience as a nurse. Notably, four studies did not specify the time period under consideration. The various types of WPV investigated in the studies include physical violence, verbal abuse, bullying, sexual harassment, and threatening behaviors. A summary of the characteristics of the included studies is provided in Table 1.

**Table 1:** Characteristics of included studies.

First author (year)	Country	Study design	Sample (female)	Mean age (*SD*)	No. facilities	Prevalence period (months)	WPV type
Musengamana (2022)	Rwanda	Cross‐sectional	195 (165)	NR	1	12	P, V, B, S
Maghraby (2020)	Egypt	Cross‐sectional	296 (All)	29 (5.3)	1	6	S
Sisawo (2017)	Gambia	Mixed‐method	219 (160)	NR	14	12	P, V, S
Mahani (2017)	South Africa	Cross‐sectional	300 (254)	NR	3	12	P, B, S, T
Weldehawaryat (2020)	Ethiopia	Cross‐sectional	348 (197)	30.7 (7.96)	19	12	P, V, B, S
Weldesenbet (2022)	Ethiopia	Cross‐sectional	339 (All)	28.0 (4.897)	4	12	S
Bekelepi (2023)	South Africa	Cross‐sectional	103 (74)	40.89 (10.36)	3	12	P, V
Abo Ali (2015)	Egypt	Cross‐sectional	430 (All)	35.9 (8.8)	1	NR	S
Boafo (2016)	Ghana	Cross‐sectional	592 (469)	31.76 (9.69)	12	12	P, V, S
Abou‐ElWafa (2015)	Egypt	Cross‐sectional	275 (257)	Emergency 33.1 (6.9); nonemergency 30.7 (8.2)	1	12	P, V, B, S
Legesse (2022)	Ethiopia	Cross‐sectional	603 (350)	NR	6	12	P, V, B, S
Ogundipe (2013)	Nigeria	Cross‐sectional	81 (57)	39.33 (9.58)	6	12	T
Abbas (2010)	Egypt	Cross‐sectional	970 (658)	NR	23	12	P, V
Kibunja (2021)	Kenya	Cross‐sectional	82 (53)	33.8 (6.8)	1	12	P, V, S, T
Samir (2012)	Egypt	Cross‐sectional	416 (NR)	NR	8	6	P, S
Agbornu (2022)	Ghana	Cross‐sectional	586 (362)	29.02 (5.60)	6	12	P
Fute (2015)	Ethiopia	Cross‐sectional	642 (404)	30.23 (6)	12	6	P, V, S
Bekalu (2023)	Ethiopia	Cross‐sectional	534 (239)	30.80 (5.8)	14	12	P, V, B, S
Likassa (2017)	Ethiopia	Cross‐sectional	203 (115)	32.9 (7.98)	4	12	P, V, B, S
Wubneh (2023)	Ethiopia	Cross‐sectional	385 (219)	32 (6)	1	12	P, V, T
Hassan (2020)	Egypt	Cross‐sectional	385 (336)	27.7 (5.4)	3	Career	P, V, S
Gabr (2021)	Egypt	Cross‐sectional	970 (All)	NR	6	NR	P, V, S
Tiruneh (2016)	Ethiopia	Cross‐sectional	386 (166)	NR	3	12	P
Douglas (2019)	Nigeria	Cross‐sectional	200 (173)	NR	3	12	V, B, S, T
Banda (2016)	Malawi	Cross‐sectional	112 (86)	NR	5	12	P, V, S, T
Tollstern Landin (2020)	Tanzania	Cross‐sectional	97 (NR)	NR	1	NR	S
Joubert (2005)	South Africa	Cross‐sectional	82 (79)	NR	1	NR	V

*Note*: B = bullying; NR = not reported; P = physical violence; V = verbal abuse; S = sexual harassment; T = threatening behavior.

Abbreviation: WPV, workplace violence.

### 3.3 Prevalence of WPV and its subtypes

The meta‐analysis exclusively considered studies that examined the prevalence of WPV based on nurses’ experience within the preceding 6–12 months. This meticulous selection aimed to mitigate the influence of recall bias, as nurses may tend to more accurately recall and report WPV incidents that have occurred in a relatively recent time frame.

**Figure 2:**
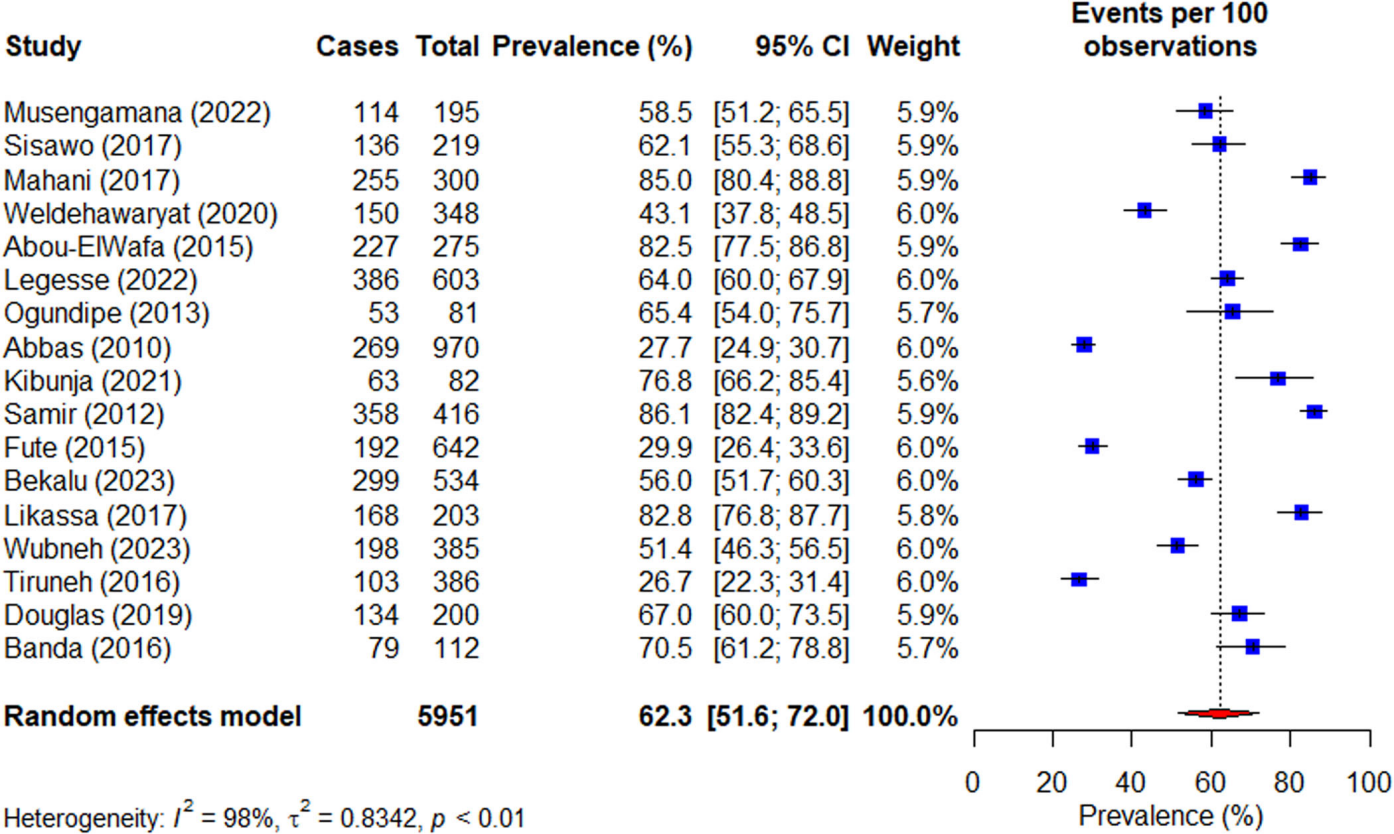
Forest plot for the prevalence of any type of WPV. CI, confidence interval; WPV, workplace violence.

As shown in Figure 2, 17 studies were included in the analysis to estimate the prevalence of any type of WPV. The pooled prevalence of WPV recorded 62.3% (95% CI: 51.6–72.0), with a significantly high heterogeneity identified among the studies (*I* ^2^ = 98%, *p* < 0.01). In terms of specific types of WPV (Figures S1–S5), verbal abuse emerged as the most prevalent, with a rate of 51.2% (95% CI: 41.3–61.1) across 15 studies. This was followed by a 23.3% (95% CI: 6.5–57.2) prevalence rate for threat (*n* = 6) and 22.9% (95% CI: 14.0–35.2) for bullying (*n* = 8). Additionally, physical abuse (*n* = 17) recorded 15.1% (95% CI: 11.0–20.4), while sexual harassment (*n* = 16) accounted for 10.3% (95% CI: 5.9–17. 5).

### 3.4 Subgroup and meta‐regression analysis

The prevalence of WPV varied across the geographical regions in Africa, recording highest in Southern Africa 78.8% (95% CI: 61.5–89.6) and lowest in the Eastern part 54.7% (95% CI: 42.9–65.9). Based on the period of study, a discernible decreasing trend in WPV rates was observed, with studies published before the year 2020 recording 64.4%, while studies published after 2020 recorded 57.8%. There was a notable variation in the prevalence of WPV regarding the academic qualification of nurses, number of facilities per study, and the sample size of studies. There was just a marginal difference in the rate of WPV for the duration of WPV estimate. The result of the meta‐regression analysis indicated no significant difference in the WPV prevalence rates across the subgroup analysis (*p* > 0.05). A detailed result of the subgroup analysis and meta‐regression is presented in Table 2.

**Table 2:** Subgroup analysis of WPV prevalence.

Subgroup	Studies (*n*)	Proportion (%)	95% CI	*I* ^2^(%)	Meta‐regression (*p*‐value)
Africa region					
East	9	54.7	42.9–65.9	98	Ref
West	3	64.6	60.3–68.6	0	0.51
North	3	69.0	22.8–94.4	100	0.34
Southern	2	78.8	61.5–89.6	91	0.13
Study period					0.62
Before 2020	11	64.4	47.0–78.7	99	
After 2020	6	57.8	50.3–64.9	91	
Majority of participants with degree and above					0.12
No	5	72.7	53.1–86.2	97	
Yes	6	53.4	36.2–69.8	99	
No. facilities					0.28
Less than 5	8	68.2	51.4–81.3	98	
5 and above	9	56.8	42.6–70.0	99	
Sample size					0.057
<300	8	71.4	63.8–78.0	88	
≥300	9	53.6	38.9–67.6	99	
Duration of WPV estimate					0.96
6 months	2	61.8	10.6–95.7	100	
12 months	15	62.4	51.8–71.9	98	

Abbreviations: CI, confidence interval; WPV, workplace violence.

### 3.5 Publication bias

**Figure 3:**
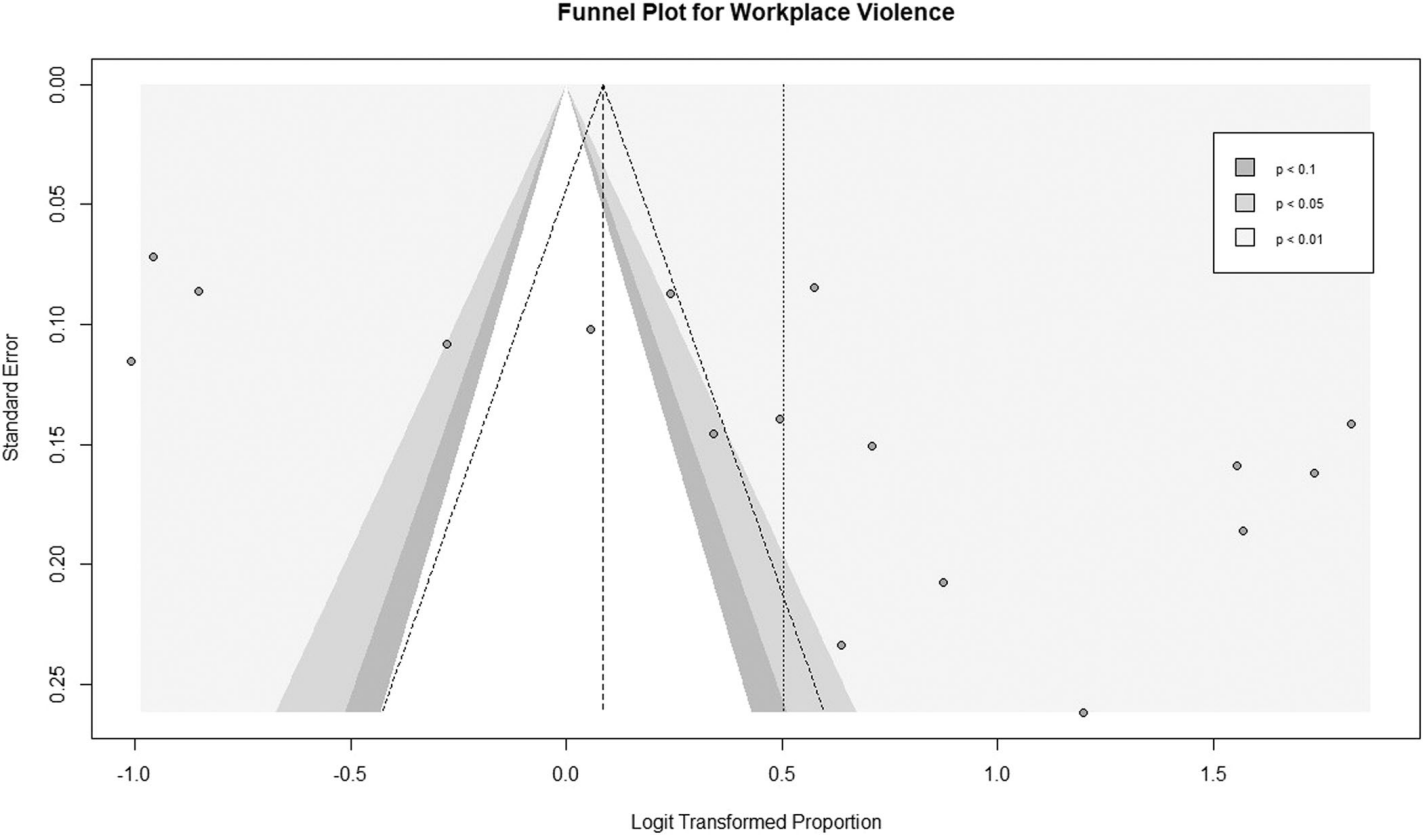
Funnel plot for the prevalence of any type of violence.

There was an asymmetrical distribution of the funnel plot for the studies that assessed the prevalence of any form of WPV (Figure 3). Statistical evidence of publication bias was further confirmed with Egger's test with *p*‐value of <0.05.

### 3.6 Predictors of WPV

Six studies assessed the predictors of WPV against nurses.^21^, ^22^, ^23^, ^24^, ^25^, ^27^ A total of 16 factors were identified to significantly predict the nurses’ experience of WPV. Emerging under four broad categories, they include demographic factors, personal habits, workplace characteristics, and nurses’ past experience with WPV (Table 3).

**Table 3:** Factors associated with WPV.

Themes	Predictors of WPV
Demographic	Marital status Gender Age
Personal habits	Intake of alcohol Chewing of chat (khat)
Workplace characteristics	Years of working experience Sex of patient frequently treated Clinical unit of practice Period of working shift Number of staff on a shift Patient waiting time Existence or awareness of institutional policy on WPV Existence or awareness of reporting procedure of WPV
Past experience	Past exposure to WPV Witness of WPV incidence Worry of WPV

Abbreviation: WPV, workplace violence.

#### 3.6.1 Demographic factors

The demographic factors associated with WPV include marital status, gender, and age. Two studies identified marital status as a predictor of WPV, with the results indicating that nurses who are single are more likely to experience WPV.^21^, ^27^ With respect to gender, the results of three studies reveals that female nurses are more likely to experience WPV as compared to males. An inconsistent pattern was observed from the available literature with respect to age as a predictor of WPV. Of fours studies that identified age as a predictor of WPV, the results of two studies found that the odds of WPV was higher for nurses of younger age,^24^ while the other studies also reported a significant association with older age.^23^, ^27^

#### 3.6.2 Personal habits

The personal habits demonstrated good evidence of an association with WPV as the results were consistent across studies. Two studies identified a significant association between alcohol intake and WPV.^21^, ^23^ The results showed that nurses who take alcohol are more likely to experience WPV. In the same studies, it was also reported that nurses who chew chat (khat) are more likely to experience WPV.

#### 3.6.3 Workplace characteristics

Several workplace‐related factors were identified to be associated with WPV. In a study, it was reported that nurses with less work experience had a higher odd of WPV.^24^ The results of two studies also found that nurses who frequently cared for male patients are more likely to experience WPV. Nurses working in inpatient units were found to have a higher odd of WPV.^24^, ^25^ Within specific units, there were a higher likelihood of WPV for nurses in the emergency,^22^, ^24^ medical, surgical, and psychiatric units.^22^ Factors such as a long waiting time of patients,^25^ and nurses’ unawareness of institutional policy and reporting procedures of WPV were significantly associated with WPV.^22^ Additionally, nurses working on regular shift had a higher odd of WPV compared to those working on night shift. In a study, it was identified that the number of staff on a shift predicted the exposure to WPV, with the result indicating a higher odd of WPV when there were five staff or less on a shift.^27^

#### 3.6.4 Past experience

Two studies identified elements of nurses past experience as a predictor of WPV. The findings of these studies revealed that nurses with past exposure to WPV had a lower risk of future incidents.^27^ In the other hand, nurses who have witnessed violence had an increased risk of experiencing it themselves. Furthermore, nurses who are worried about WPV had a higher odd of its exposure.^22^

## 4 DISCUSSION

The issue of WPV against nurses has garnered significant research focus. However, the narrative surrounding this topic within the context of Africa remains relatively underexplored. This systematic review marks a pioneering effort to provide comprehensive evidence regarding the extent of WPV against nurses in Africa.

The result of our meta‐analysis revealed that the pooled prevalence of WPV was 62.3%. Although the sub‐group analysis revealed a varying prevalence rate across the geographical areas in Africa, these differences were not significant, suggesting that WPV is concerning and pervasive issue that transcends geographical barriers in Africa. Compared to existing meta‐analysis on WPV, our prevalence estimate is higher. Notably, in a global meta‐analysis, the prevalence of WPV recorded 59.2% among nurses.^4^ In South‐East Asian and Western Pacific Regions, recent meta‐analysis has indicated a WPV prevalence rate of 58%.^47^ There are several possible explanations to this disparity. First, many African healthcare systems struggle with limited resources, overcrowded facilities, and understaffing, which can create a stressful environment for both patients and healthcare workers.^38^ These challenging conditions may contribute to heightened tensions and frustration, increasing the potential for violent incidents. Additionally, cultural norms and societal attitudes toward healthcare workers can also differ in African regions. In some cases, nurses may face disrespect or hostility due to deeply ingrained beliefs or misunderstandings about their roles. In terms of specific types of WPV, our findings revealed a prevalence rate highest for verbal abuse and lowest for sexual harassment. Similar pattern has been observed in other studies.^4^, ^47^, ^48^ The elevated occurrence of verbal abuse could be attributed to its relative ease of perpetration.^21^ Furthermore, the absence of immediate physical harm or injury associated with verbal abuse might lead individuals to downplay its severity, potentially fostering a higher level of tolerance.

Several factors were identified to significantly predict the occurrence of WPV, encompassing demographic factors, personal habits, workplace characteristics, and nurses’ past experience with WPV. Among these, gender, marital status, alcohol consumption, and chewing khat consistently appeared to predict WPV exposure across multiple studies. Notably, in terms of gender comparison, we found that females were more likely to experience WPV than their male counterparts. However, in other regions, a mixed pattern of gender‐related WPV prevalence is observed.^8^, ^9^ Our findings may be due to traditional gender roles and expectations in Africa, where individuals hold the view that men are at the top of the hierarchical structure and are superior to female. Resonating to previous meta‐analysis,^4^ we found that nurses who are single or not married are more prone to WPV. It is likely that single individuals might face different social dynamics that could influence their vulnerability.^49^ Moreover, married nurses may have a stronger support system which could contribute to a reduced risk of exposure to WPV. Furthermore, our study revealed that nurses’ personal habits, such as alcohol consumption and chat chewing, heightened the likelihood of WPV. It possible that the stimulant effects of khat and the depressant effects of alcohol can compromise a nurse's ability to provide optimal care, potentially leading to negative reactions from patients and their families.

This review sheds important insights on the issue of WPV in the context of Africa. However, there are some limitations that should be acknowledged. First, the studies analyzed exhibited a high level of heterogeneity. Moreover, there was a significant publication bias among the included studies, potentially resulting in an underestimated prevalence estimate. Furthermore, the exclusion of non‐English language articles may overlook perspectives from other linguistic groups, thereby limiting the generalizability of the findings.

### 4.1 Implications of findings

The high prevalence of WPV against nurses in Africa poses multifaceted implications. Beyond the immediate impact on the mental and physical well‐being of nurses, there is a potential degradation in the quality of patient care as healthcare providers grapple with stress and safety concerns. This could trigger significant challenges in staff retention and recruitment, leading to a shortage of skilled professionals and straining healthcare systems. Addressing this issue necessitates a concerted effort to create a safer and more supportive environment. First, establishing clear reporting mechanisms and support systems for victims is crucial, creating an environment where incidents are promptly addressed. Additionally, fostering a culture of respect within healthcare institutions through awareness campaigns and leadership initiatives can contribute to a positive and safe workplace. Policymakers should also play a role by enacting and enforcing legislation that specifically addresses WPV in the healthcare sector.

## 5 CONCLUSION

This review illuminates the pervasive issue of WPV against nurses in Africa, highlighting a concerning prevalence rate of 62.3%. This underscores the need for targeted interventions and policy measures to ensure safer workplace environments for nurses in Africa.

## AUTHOR CONTRIBUTIONS

**Emmanuel Ekpor**: conceptualization; investigation; methodology; validation; software; formal analysis; project administration; data curation; writing—original draft; writing—review & editing; visualization; resources. **Emmanuel Kobiah**: data curation; validation; writing—original draft; writing—review & editing; investigation; project administration. **Samuel Akyirem**: methodology; validation; software; data curation; supervision; formal analysis; visualization; writing—review & editing; writing—original draft.

## CONFLICT OF INTEREST STATEMENT

The authors declare no conflicts of interest.

## TRANSPARENCY STATEMENT

The lead author Emmanuel Ekpor affirms that this manuscript is an honest, accurate, and transparent account of the study being reported; that no important aspects of the study have been omitted; and that any discrepancies from the study as planned (and, if relevant, registered) have been explained.

## Supporting information

Supporting information.

Supporting information.

Supporting information.

## Data Availability

The data that supports the findings of this study are available in the manuscript and supplementary material of this article.
