# Supplementary material for: Prevalence and predictors of workplace violence against nurses in Africa: A systematic review and meta‐analysis
Source: Health Sci Rep. 2024 Apr 21;7(4):e2068. doi: 10.1002/hsr2.2068 (PMC11033334; doi:10.1002/hsr2.2068)
Supplement: Supplementary file 1 — Supporting information. [file HSR2-7-e2068-s001.docx]

**Supplementary figure 1-5**


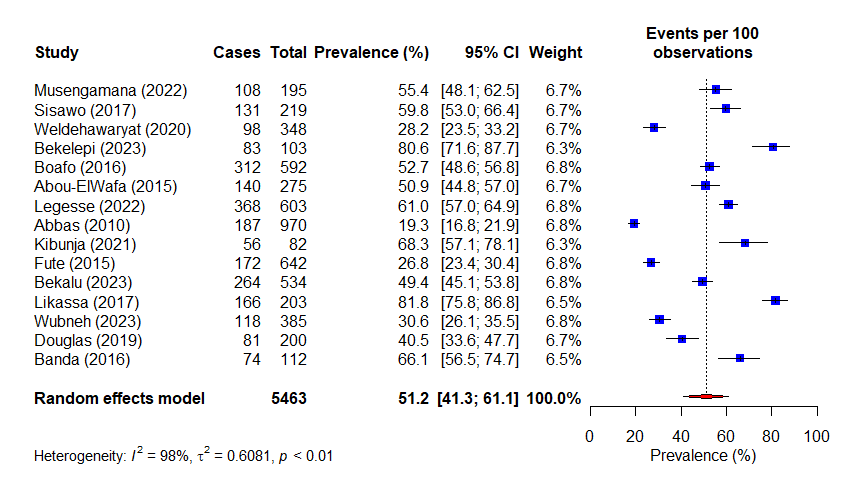


**Figure S1.** Forest plot for prevalence of verbal abuse


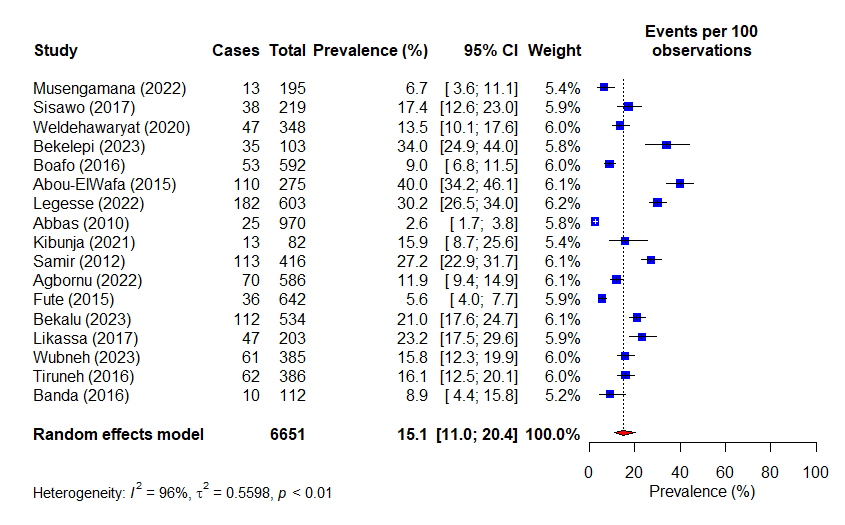


**Figure S2.** Forest plot for prevalence of physical abuse


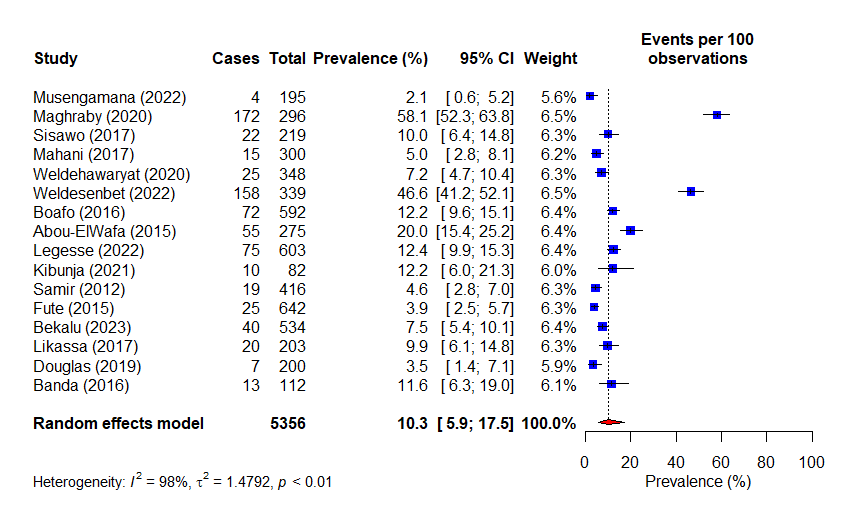


**Figure S3.** Forest plot for the prevalence of sexual harassment


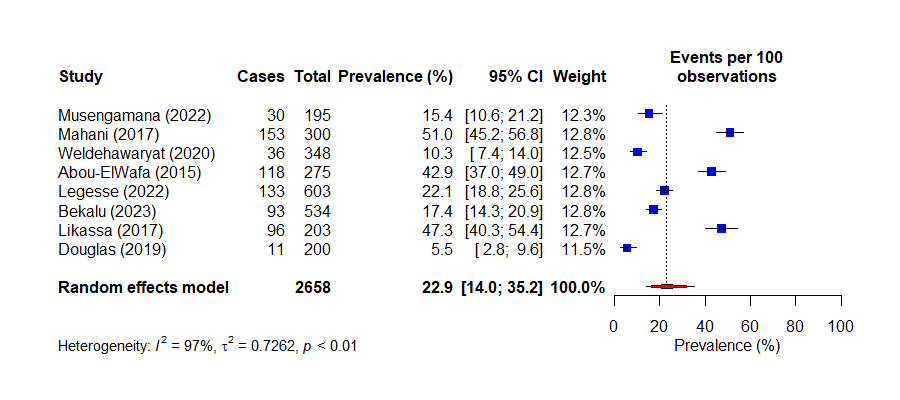


**Figure S4.** Forest plot for the prevalence of bullying


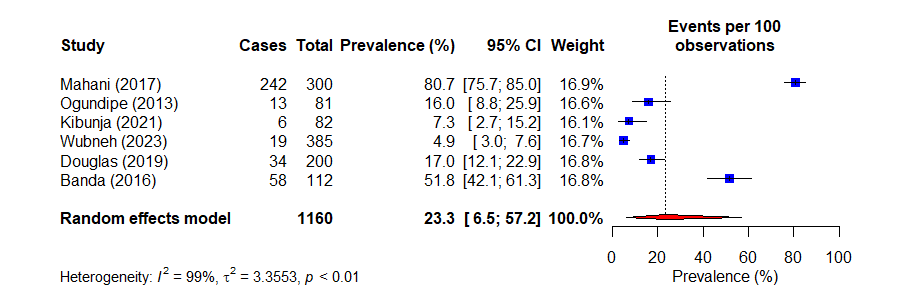


**Figure S5.** Forest plot for the prevalence of threat
