# Supplementary material for: Prevalence and predictors of workplace violence against nurses in Africa: A systematic review and meta‐analysis
Source: Health Sci Rep. 2024 Apr 21;7(4):e2068. doi: 10.1002/hsr2.2068 (PMC11033334; doi:10.1002/hsr2.2068)
Supplement: Supplementary file 2 — Supporting information. [file HSR2-7-e2068-s002.docx]

**Supplementary table 1.** Database search strategy

Medline

| Concept | Search string |
| --- | --- |
| #1  Workplace violence | exp Workplace Violence/ OR exp Violence/ OR "workplace violence".ti,ab. OR "lateral violence".ti,ab. OR "horizontal violence".ti,ab. OR violence.ti,ab. OR "Sexual Harassment".ti,ab. OR "sexual abuse".ti,ab. OR assault.ti,ab. OR "assaultive behavior".ti,ab. OR abuse.ti,ab. OR aggression.ti,ab. OR bullying.ti,ab. OR harassment.ti,ab. OR incivility.ti,ab. OR WPV.ti,ab. |
| #2  Nurse | exp Nurses/ OR exp Nursing Staff, Hospital/ OR exp Nursing Staff/ OR Nursing/ OR Nurse*.ti,ab. OR Nursing.ti,ab. OR "Registered Nurse".ti,ab. OR "Professional Nurse".ti,ab. OR "Staff Nurse".ti,ab. |
| #3  Africa | exp Africa/ OR Africa.ti,ab OR Algeria.ti,ab OR Angola.ti,ab OR Benin.ti,ab OR Botswana.ti,ab OR "Burkina Faso".ti,ab OR Burundi.ti,ab OR "Cabo Verde".ti,ab OR Cameroon.ti,ab OR "Central African Republic".ti,ab OR Chad.ti,ab OR Comoros.ti,ab OR "Democratic Republic of the Congo".ti,ab OR "Republic of the Congo".ti,ab OR "Cote d'Ivoire".ti,ab OR Djibouti.ti,ab OR Egypt.ti,ab OR "Equatorial Guinea".ti,ab OR Eritrea.ti,ab OR Eswatini.ti,ab OR Ethiopia.ti,ab OR Gabon.ti,ab OR Gambia.ti,ab OR Ghana.ti,ab OR Guinea.ti,ab OR Guinea-Bissau.ti,ab OR Kenya.ti,ab OR Lesotho.ti,ab OR Liberia.ti,ab OR Libya.ti,ab OR Madagascar.ti,ab OR Malawi.ti,ab OR Mali.ti,ab OR Mauritania.ti,ab OR Mauritius.ti,ab OR Morocco.ti,ab OR Mozambique.ti,ab OR Namibia.ti,ab OR Niger.ti,ab OR Nigeria.ti,ab OR Rwanda.ti,ab OR "Sao Tome and Principe".ti,ab OR Senegal.ti,ab OR Seychelles.ti,ab OR "Sierra Leone".ti,ab OR Somalia.ti,ab OR "South Africa".ti,ab OR "South Sudan".ti,ab OR Sudan.ti,ab OR Tanzania.ti,ab OR Togo.ti,ab OR Tunisia.ti,ab OR Uganda.ti,ab OR Zambia.ti,ab OR Zimbabwe.ti,ab |
| #4 | #1 AND #2 AND #3 |
| #5 | #1 AND #2 AND #3 limit to yr="2000 - 2023" |

CINAHL

| Concept | Search string |
| --- | --- |
| #1  Workplace violence | (MH "Workplace Violence") OR (MH "Violence+") OR TI("Work place violence" OR "lateral violence" OR "horizontal violence" OR violence OR "Sexual Harassment" OR "sexual abuse" OR sexual OR assault OR "assaultive behavior" OR abuse OR aggression OR bullying OR harassment OR incivility OR WPV) OR AB("Work place violence" OR "lateral violence" OR "horizontal violence" OR violence OR "Sexual Harassment" OR "sexual abuse" OR assault OR "assaultive behavior" OR abuse OR aggression OR bullying OR harassment OR incivility OR WPV) |
| #2  Nurse | (MH "Nurses+") OR (MH "Nursing Staff, Hospital") OR (MH "Staff Nurses") OR (MH "Registries, Personnel") OR TI(Nurse* OR Nursing OR "Registered Nurse" OR "Professional Nurse" OR "Staff Nurse") OR AB(Nurse* OR Nursing OR "Registered Nurse" OR "Professional Nurse" OR "Staff Nurse") |
| #3  Africa | (MH "Africa+") OR Africa OR Algeria OR Angola OR Benin OR Botswana OR "Burkina Faso" OR Burundi OR "Cabo Verde" OR Cameroon OR "Central African Republic" OR Chad OR Comoros OR "Democratic Republic of the Congo" OR "Republic of the Congo" OR "Cote d'Ivoire" OR Djibouti OR Egypt OR "Equatorial Guinea" OR Eritrea OR Eswatini OR Ethiopia OR Gabon OR Gambia OR Ghana OR Guinea OR Guinea-Bissau OR Kenya OR Lesotho OR Liberia OR Libya OR Madagascar OR Malawi OR Mali OR Mauritania OR Mauritius OR Morocco OR Mozambique OR Namibia OR Niger OR Nigeria OR Rwanda OR "Sao Tome and Principe" OR Senegal OR Seychelles OR "Sierra Leone" OR Somalia OR "South Africa" OR "South Sudan" OR Sudan OR Tanzania OR Togo OR Tunisia OR Uganda OR Zambia OR Zimbabwe |
| #4 | #1 AND #2 AND #3 |
| #5 | #1 AND #2 AND #3 limit to yr="2000 - 2023" |

PsycINFO

| Concept | Search string |
| --- | --- |
| #1  Workplace violence | (exp Workplace Violence/ or exp Violence/) OR "workplace violence".ti,ab. OR "lateral violence".ti,ab. OR "horizontal violence".ti,ab. OR violence.ti,ab. OR "Sexual Harassment".ti,ab. OR "sexual abuse".ti,ab. OR assault.ti,ab. OR "assaultive behavior".ti,ab. OR abuse.ti,ab. OR aggression.ti,ab. OR bullying.ti,ab. OR harassment.ti,ab. OR incivility.ti,ab. OR WPV.ti,ab. |
| #2  Nurse | exp Nurses/ OR exp Nursing/ OR Nurse*.ti,ab. OR Nursing.ti,ab. OR "Registered Nurse".ti,ab. OR "Professional Nurse".ti,ab. OR "Staff Nurse".ti,ab. |
| #3  Africa | Africa.ti,ab OR Algeria.ti,ab OR Angola.ti,ab OR Benin.ti,ab OR Botswana.ti,ab OR "Burkina Faso".ti,ab OR Burundi.ti,ab OR "Cabo Verde".ti,ab OR Cameroon.ti,ab OR "Central African Republic".ti,ab OR Chad.ti,ab OR Comoros.ti,ab OR "Democratic Republic of the Congo".ti,ab OR "Republic of the Congo".ti,ab OR "Cote d'Ivoire".ti,ab OR Djibouti.ti,ab OR Egypt.ti,ab OR "Equatorial Guinea".ti,ab OR Eritrea.ti,ab OR Eswatini.ti,ab OR Ethiopia.ti,ab OR Gabon.ti,ab OR Gambia.ti,ab OR Ghana.ti,ab OR Guinea.ti,ab OR Guinea-Bissau.ti,ab OR Kenya.ti,ab OR Lesotho.ti,ab OR Liberia.ti,ab OR Libya.ti,ab OR Madagascar.ti,ab OR Malawi.ti,ab OR Mali.ti,ab OR Mauritania.ti,ab OR Mauritius.ti,ab OR Morocco.ti,ab OR Mozambique.ti,ab OR Namibia.ti,ab OR Niger.ti,ab OR Nigeria.ti,ab OR Rwanda.ti,ab OR "Sao Tome and Principe".ti,ab OR Senegal.ti,ab OR Seychelles.ti,ab OR "Sierra Leone".ti,ab OR Somalia.ti,ab OR "South Africa".ti,ab OR "South Sudan".ti,ab OR Sudan.ti,ab OR Tanzania.ti,ab OR Togo.ti,ab OR Tunisia.ti,ab OR Uganda.ti,ab OR Zambia.ti,ab OR Zimbabwe.ti,ab |
| #4 | #1 AND #2 AND #3 |
| #5 | #1 AND #2 AND #3 limit to yr="2000 - 2023" |

Scopus

| Concept | Search string |
| --- | --- |
| #1  Workplace violence | TITLE-ABS-KEY ("Work place violence" OR "lateral violence" OR "horizontal violence" OR violence OR "Sexual Harassment" OR "sexual abuse" OR assault OR “assaultive behavior” OR abuse OR aggression OR bullying OR harassment OR incivility OR WPV) |
| #2  Nurse | TITLE-ABS-KEY (Nurse* OR Nursing OR "Registered Nurse" OR "Professional Nurse" OR "Staff Nurse") |
| #3  Africa | TITLE-ABS-KEY ("Africa" OR "Algeria" OR "Angola" OR "Benin" OR "Botswana" OR "Burkina Faso" OR "Burundi" OR "Cabo Verde" OR "Cameroon" OR "Central African Republic" OR "Chad" OR "Comoros" OR "Democratic Republic of the Congo" OR "Republic of the Congo" OR "Cote d'Ivoire" OR "Djibouti" OR "Egypt" OR "Equatorial Guinea" OR "Eritrea" OR "Eswatini" OR "Ethiopia" OR "Gabon" OR "Gambia" OR "Ghana" OR "Guinea" OR "Guinea-Bissau" OR "Kenya" OR "Lesotho" OR "Liberia" OR "Libya" OR "Madagascar" OR "Malawi" OR "Mali" OR "Mauritania" OR "Mauritius" OR "Morocco" OR "Mozambique" OR "Namibia" OR "Niger" OR "Nigeria" OR "Rwanda" OR "Sao Tome and Principe" OR "Senegal" OR "Seychelles" OR "Sierra Leone" OR "Somalia" OR "South Africa" OR "South Sudan" OR "Sudan" OR "Tanzania" OR "Togo" OR "Tunisia" OR "Uganda" OR "Zambia" OR "Zimbabwe") |
| #4 | #1 AND #2 AND #3 |
| #5 | #1 AND #2 AND #3 limit to yr="2000 - 2023" |
