# Supplementary material for: Prevalence and predictors of workplace violence against nurses in Africa: A systematic review and meta‐analysis
Source: Health Sci Rep. 2024 Apr 21;7(4):e2068. doi: 10.1002/hsr2.2068 (PMC11033334; doi:10.1002/hsr2.2068)
Supplement: Supplementary file 3 — Supporting information. [file HSR2-7-e2068-s003.docx]

**Supplementary table 2.** JBI Checklist for Prevalence Studies

| First author (year) | Was the sample frame appropriate to address the target population? | Were study participants sampled in an appropriate way? | Was the sample size adequate? | Were the study subjects and the setting described in detail? | Was the data analysis conducted with sufficient coverage of the identified sample? | Were valid methods used for the identification of the condition? | Was the condition measured in a standard, reliable way for all participants? | Was there appropriate statistical analysis? | Was the response rate adequate, and if not, was the low response rate managed appropriately? | Score |
| --- | --- | --- | --- | --- | --- | --- | --- | --- | --- | --- |
| Musengamana (2022) | Yes | Yes | Yes | Yes | Yes | No | Unclear | Yes | Yes | 7 |
| Maghraby (2020) | Yes | Yes | Yes | Yes | Yes | No | Unclear | Yes | Yes | 7 |
| Sisawo (2017) | Yes | Unclear | Yes | Yes | Yes | Yes | Yes | Yes | Yes | 8 |
| Mahani (2017) | Yes | Yes | No | No | Yes | No | Yes | Unclear | Yes | 5 |
| Weldehawaryat (2020) | Yes | Yes | Yes | Yes | Yes | Yes | Yes | Yes | Yes | 9 |
| Weldesenbet (2022) | Yes | Yes | Yes | Yes | Yes | Unclear | Yes | Yes | Yes | 8 |
| Bekelepi (2023) | Yes | Yes | No | Yes | Yes | Yes | Unclear | Yes | Yes | 7 |
| Abo Ali (2015) | No | Yes | Yes | Yes | Yes | Yes | Unclear | Yes | Yes | 7 |
| Boafo (2016) | Yes | Yes | Unclear | Yes | Yes | Yes | Unclear | Yes | Unclear | 6 |
| Abou-ElWafa (2015) | No | Unclear | No | Yes | Yes | Yes | Unclear | Yes | Yes | 5 |
| Legesse (2022) | Yes | Yes | Yes | Yes | Yes | Yes | Yes | Yes | Yes | 9 |
| Ogundipe (2013) | No | Unclear | No | Yes | Yes | No | No | Unclear | Yes | 3 |
| Abbas (2010) | Yes | Unclear | Yes | Yes | Yes | Yes | Unclear | Yes | No | 6 |
| Kibunja (2021) | No | Yes | Yes | Yes | Yes | Yes | Unclear | Yes | Yes | 7 |
| Samir (2012) | Yes | Yes | Yes | Yes | Yes | Yes | No | Yes | Yes | 8 |
| Agbornu (2022) | Yes | Yes | Yes | Yes | Yes | Yes | Unclear | Yes | Yes | 8 |
| Fute (2015) | Yes | Yes | Yes | Yes | Yes | Yes | Unclear | Yes | Yes | 8 |
| First author (year) | Was the sample frame appropriate to address the target population? | Were study participants sampled in an appropriate way? | Was the sample size adequate? | Were the study subjects and the setting described in detail? | Was the data analysis conducted with sufficient coverage of the identified sample? | Were valid methods used for the identification of the condition? | Was the condition measured in a standard, reliable way for all participants? | Was there appropriate statistical analysis? | Was the response rate adequate, and if not, was the low response rate managed appropriately? | Score |
| Bekalu (2023) | Yes | Yes | Yes | Yes | Yes | Yes | Yes | Yes | Yes | 9 |
| Likassa (2017) | Yes | Yes | Yes | Yes | Yes | Yes | Yes | Yes | Yes | 9 |
| Wubneh (2023) | Yes | Yes | Yes | Yes | Yes | Yes | Yes | Yes | Yes | 9 |
| Hassan (2020) | Yes | No | Unclear | No | Yes | No | Unclear | Yes | Unclear | 3 |
| Gabr (2021) | Yes | Yes | Unclear | Yes | Yes | Yes | Unclear | Yes | Unclear | 6 |
| Tiruneh (2016) | Yes | Unclear | Yes | Yes | Yes | Yes | Unclear | Yes | Yes | 7 |
| Douglas (2019) | Yes | Yes | Unclear | Yes | Yes | No | No | Unclear | Yes | 5 |
| Banda (2016) | Yes | No | Unclear | Yes | Yes | No | Unclear | Yes | No | 4 |
| Tollstern Landin (2020) | No | Unclear | Yes | Yes | Yes | No | Unclear | Yes | Yes | 5 |
| Joubert (2005) | Yes | Unclear | Unclear | No | Yes | Yes | Unclear | Unclear | No | 3 |
